# Maternal Diet-induced Obesity Programs Cardiovascular Dysfunction in Adult Male Mouse Offspring Independent of Current Body Weight

**DOI:** 10.1210/en.2014-1383

**Published:** 2014-07-22

**Authors:** Heather L. Blackmore, Youguo Niu, Denise S. Fernandez-Twinn, Jane L. Tarry-Adkins, Dino A. Giussani, Susan E. Ozanne

**Affiliations:** University of Cambridge, Metabolic Research Laboratories and MRC Metabolic Diseases Unit, Wellcome Trust-MRC Institute of Metabolic Science (H.L.B., D.S.F.-T., J.L.T.-A., S.E.O.), Addenbrookes Hospital, Cambridge CB2 0QQ, United Kingdom; and Department of Physiology, Development, and Neuroscience (Y.N., D.A.G.), University of Cambridge, Cambridge CB2 3EG, United Kingdom

## Abstract

Obese pregnancies are not only associated with adverse consequences for the mother but also the long-term health of her child. Human studies have shown that individuals from obese mothers are at increased risk of premature death from cardiovascular disease (CVD), but are unable to define causality. This study aimed to determine causality using a mouse model of maternal diet–induced obesity. Obesity was induced in female C57BL/6 mice by feeding a diet rich in simple sugars and saturated fat 6 weeks prior to pregnancy and throughout pregnancy and lactation. Control females were fed laboratory chow. Male offspring from both groups were weaned onto chow and studied at 3, 5, 8, and 12 weeks of age for gross cardiac morphometry using stereology, cardiomyocyte cell area by histology, and cardiac fetal gene expression using qRT-PCR. Cardiac function was assessed by isolated Langendorff technology at 12 weeks of age and hearts were analyzed at the protein level for the expression of the β1 adrenergic receptor, muscarinic type-2 acetylcholine receptor, and proteins involved in cardiac contraction. Offspring from obese mothers develop pathologic cardiac hypertrophy associated with re-expression of cardiac fetal genes. By young adulthood these offspring developed severe systolic and diastolic dysfunction and cardiac sympathetic dominance. Importantly, cardiac dysfunction occurred in the absence of any change in corresponding body weight and despite the offspring eating a healthy low-fat diet. These findings provide a causal link to explain human observations relating maternal obesity with premature death from CVD in her offspring.

The growing prevalence of obesity is posing a significant burden for future generations by increasing their risk of noncommunicable diseases such as cardiovascular disease (CVD). Furthermore, obesity prevalence is increasing globally including women of childbearing age (1). Although obesity during pregnancy is associated with immediate adverse pregnancy and neonatal outcomes (2, 3), growing evidence from the field of Developmental Origins of Health and Disease suggests that early life insults (eg, obesity, under nutrition, hypoxia), shape our vulnerability to disease (4, 5). There is strong indication from human studies and animal models that exposure to obesity in utero and early postnatal life increases risk of later CVD. For example, in a recent study of 37 709 individuals, maternal obesity was associated with increased all-cause mortality and admission to hospital for cardiovascular events in her offspring (6). Similar observations were made in a smaller Finnish cohort where maternal body mass index was associated with an increased risk of coronary heart disease in the offspring (7).

Unfortunately, confounding factors in human studies limit any inferences from such studies to pure association, and this is where animal models of maternal overnutrition and obesity have been invaluable in determining mechanisms underlying programming. These models include manipulation of the maternal diet such that it contains either high fat (8–11), or is rich in both sugar and fat (12, 13), overfeeding by raising the recommended food intake (14, 15), and genetic models of obesity (16). Exposure of offspring to an overnourished and/or obesogenic environment in early life has been associated with later obesity (8), endothelial dysfunction (10, 17, 18), elevated cholesterol (19), hypertension (8, 9, 12, 19, 20), and cardiac hypertrophy (13, 18) in young adults. Whereas a number of studies have shown that exposure to maternal overnutrition and/or obesity programs an increased risk of CVD, as well as obesity (8, 9, 12), the effect of such exposures on offspring adult cardiac structure and function independent of the offspring themselves developing obesity has not been addressed. Because obesity itself is an independent risk factor for CVD (21, 22), tackling this question may elucidate mechanisms by which CVD is programmed in the offspring of obese pregnancy via alternative pathways, thereby identifying novel therapeutic targets for intervention. We investigated this in a well-established model of maternal diet-induced obesity (13). The development of cardiac hypertrophy was previously demonstrated at 8 weeks of age in male offspring (13). Therefore, using an integrated approach of assessing both cardiac structure and molecular markers of cardiac hypertrophy, male offspring exposed to maternal diet-induced obesity were studied at 3, 5, 8, and 12 weeks of age. Importantly, molecular and structural changes were identified within the context of developing impairments in cardiac function using the isolated Langendorff heart perfusion preparation.

## Materials and Methods

### Animal model

All studies were approved by the local ethics committee of the University of Cambridge and were conducted according to the Home Office Animals (Scientific Procedures) Act 1986 (United Kingdom). At 3 weeks of age female C57BL/6J mice were placed onto either an ad libitum control chow diet (RM1) [7% simple sugars, 3% fat (wt/wt) (Special Dietary Services)] or a highly palatable energy-rich obesogenic diet [10% simple sugars, 20% animal lard (wt/wt) (Special Dietary Services)] supplemented with sweetened condensed milk [approximately 55% simple sugars, 8% fat, (wt/wt) (Nestle)] contained in a pot within the female cage, and vitamin and mineral mix AIN93G (Special Dietary Services) for 6 weeks before mating. Breeding was performed in house. A detailed breakdown of the dietary composition has been previously reported (12). Offspring from the first pregnancy were culled at weaning, providing proven breeder status. Females fed the obesogenic diet were mated for second pregnancy once reaching 10 g absolute fat mass, confirming prepregnancy obesity (Minispec Time Domain Nuclear Resonance, Bruker Optics). On reaching 10 g fat mass or 1 week of rest for control females, proven breeders were remated for second pregnancy and maintained on their respective diets during pregnancy and lactation. Dams were weighed at the beginning and end of pregnancy and at the end of lactation. Litter size was standardized at random to six pups 48 hours post delivery, maintaining an equal sex ratio where possible. Female offspring were culled at weaning with males being studied at 3, 5, 8, and 12 weeks of age. In total there were 31 litters from control females and 38 from obese females. Offspring from obese dams are labeled as offspring of obese dams (OffOb). Two littermates were used at each time point; the heart from one animal was frozen and the other animal's heart was fixed in 10% neutral buffered formalin (NBF). Offspring culled at day 22 (3 weeks old) were weaned from their respective dam and fasted overnight. Offspring used for later time points (5, 8, and 12 weeks of age) were weaned ad libitium onto standard laboratory chow [RM1 (Special Dietary Services)]. At 3, 5, and 8 weeks of age mice were culled after an overnight fast by carbon dioxide asphyxiation. Due to the requirements of the isolated Langendorff heart perfusion, offspring at 12 weeks of age remained fed and were culled by cervical dislocation. The hearts used in the Langendorff heart perfusion were fixed post procedure, whereas the heart of its littermate was frozen.

Blood was allowed to clot and then centrifuged at 7200 g (Eppendorff centrifuge 5424) for 3 minutes and the serum removed. To enhance the yield, the supernatant was respun under the same conditions and serum removed and frozen at −80 C until analysis. Total cholesterol, triglycerides, and free fatty acids were measured at the Core Biochemical Assay Laboratory (Addenbrookes Hospital).

### Cardiac stereology

Offspring hearts from all time points were fixed in 10% NBF, processed, and embedded in paraffin. Serial sections were cut in the coronal plane at 10 μm using a microtome (Leica Microsystems). Slides were processed and stained with hematoxylin and eosin. Sections were visualized using a BX-50 microscope (Olympus) fitted with a motorized specimen stage and microcator. Analyses were performed blinded using the Computer Assisted Stereology Toolbox version 2.0 (Olympus). Wall and compartmental volumes (mm^3^) were calculated for the left ventricle (LV) [(LV) combining the LV free wall and interventricular septum and right ventricle (RV)] using the Cavalieri principle. Detailed methodology has been described previously (13, 23).

### Cardiomyocyte cross-sectional cell area

Wheat germ agglutinin was used to stain cardiomyocyte cell borders at all time points using a previously described method [Texas Red-X conjugate, (Molecular Probes, Life Technologies)] (13). Hearts were mounted onto a chuck in the orientation of the heart in vivo using Tissue Tek optimal cutting temperature compound (Sakura). The heart was sectioned in the coronal plane at −21 C until reaching the mid-cardiac region. Two 7-μm sections were cut consecutively and mounted onto a charged slide. Sections were fixed in 10% NBF for 20 minutes at room temperature and washed with phosphate buffered saline (PBS) before incubating with 10 μg/ml of conjugated agglutinin while gently rocking in darkness for 2 hours at room temperature. Slides were then washed with PBS and air-dried before addition of Vectashield mounting medium with 4′,6-Diamidino-2-phenylindole (Vector Laboratories). Images were analyzed double-blinded using CellD software (Olympus). Only myocytes in cross-section were selected. Area was calculated by averaging across five separate images per heart (approximately 400–600 cells measured per heart).

### Cardiac mRNA expression

At all time points, RNA was extracted from 30 mg of powdered tissue from whole heart using the mirVANA isolation kit as per the manufacturer's instructions (Life Technologies). RNA concentration (ng/ml) was quantified using the Nanodrop spectrophotometer (Nanodrop Technologies). cDNA synthesis was performed using 1 μg of RNA, oligo-dT primers, and Moloney Murine Leukemia Virus reverse transcriptase (Promega). SYBR green technology coupled with designed intron-spanning Sigma primers were used to quantify gene expression (Sigma-Aldrich). Primer sequences are listed in Supplemental Table 1. Gene expression was quantified using a Step One Plus RT-PCR machine (Applied Biosystems). mRNA expression was normalized to housekeeper *PPIA*. There was no significant effect of either maternal diet or offspring age on mRNA expression of *PPIA*.

### Isolated Langendorff heart perfusion

#### Basal cardiac function

At 12 weeks of age, fed mice were killed by cervical dislocation. After recording of body weight, a thoracotomy was performed and the heart was rapidly excised and immediately placed into ice-cold Krebs-Henseleit bicarbonate (KHB) buffer (120mM NaCl, 4.7mM KCl, 1.2mM MgSO_2_·7H_2_O, 1.2mM KH_2_PO_4_, 25mM NaHCO_3_, 10mM glucose, and 1.3mM CaCl_2_·2H_2_O). The heart was cannulated via the aorta and perfused via the coronary arteries whereas lung, mediastinal, and pericardiac brown adipose tissue were dissected away. A pulmonary arteriotomy was performed. Hearts were perfused at a constant pressure of 65 mmHg (24). A small flexible nonelastic balloon was inserted into the LV through the left atrium (LA). The balloon was filled with deionized water and attached to a rigid, deionized water-filled catheter connected to a calibrated pressure transducer (Argon Medical Devices). The volume of the balloon was adjusted by injection of deionized water with a 100-μL Hamilton syringe to get a recording of LV end diastolic pressure (LVEDP) between 5 and 10 mmHg (25).

Recirculating KHB was filtered through a 5-μm cellulose nitrate filter (Millipore) and gassed with O_2_:CO_2_ (95:5) at 37 C. After an initial stabilization period of 15 minutes, basal measurements of heart rate (HR), LV systolic pressure (LVSP) and LVEDP were recorded. Baseline LV developed pressure (LVDP) was calculated as LVSP-LVEDP. The maximum (dP/d*t*_max_) and minimum (dP/d*t*_min_) first derivatives of LV pressure were calculated automatically using the M-PAQ data acquisition system (Maastricht Programmable AcQusition System).

#### Treatment with parasympathetic and sympathetic agonists

Cardiac chronotropic and inotropic responsiveness to carbachol (carbamylcholine chloride; range, 10^−9^– 10^−7^ M, [Sigma-Aldrich]) and isoprenaline [(−)-Isoproterenol (+)-bitartrate salt; range, 10^−10^–10^−8^ M (Sigma-Aldrich)] was investigated. Carbachol and isoprenaline were dissolved in KHB and introduced into the heart via the compliance chamber of the Langendorff apparatus. The heart was perfused in a non-recirculating way to avoid accumulation of carbachol or isoprenaline within the system. Recovery time (ranging from 5–15 minutes) was allowed between each bolus to allow HR and LVDP to stabilize to baseline values before the administration of the next bolus.

### Cardiac protein expression

Proteins critical for the inotropic and chronotropic action of the heart were studied in fed tissue to establish underlying mechanisms of cardiac dysfunction at 12 weeks of age. Protein expression was determined by Western blot, as previously described (13). Protein (10 μg) was loaded for each sample. Equal protein loading was confirmed by Coomassie blue staining. Antibodies to the following proteins were used:phosphorylated cardiac troponin-I (serine 23/24); total troponin-I; ATP2A2/SERCA2; tropomyosin-1 (all Cell Signaling); β_1_ adrenergic receptor (Santa Cruz Biotechnology), Muscarinic type-2 acetylcholine receptor (Abcam). Horseradish peroxidise-linked secondary antibodies were used (Jackson ImmunoResearch). Protein bands were detected using West Pico Chemiluminescence reagent (Pierce, Thermo Scientific). Membranes were either imaged using the Bio-Rad Chemi-doc MP system and analyzed using Image Lab Software (Bio-Rad Laboratories) or exposed to photosensitive film (GE Healthcare Amersham Hyperfilm ECL [Fisher Scientific]) and analyzed using AlphaEase imaging software (Alpha Innotech). To ensure the chemiluminescent signal changed in a linear manner, the ratio between loading controls (50% and 100% pooled sample) was confirmed for each detected protein.

### Statistical analysis

Data were analyzed using Prism 5 (GraphPad). Offspring growth trajectory was plotted as an average body weight per litter from day 3–84 and all litters used were included. Data containing multiple time points were analyzed using a 2-way ANOVA, with the exception of offspring growth trajectory, which was analyzed using a repeated-measures 2-way ANOVA. Holm Sidak post hoc correction was applied in the presence of a significant (*P* < .05) overall effect of maternal diet or offspring age. When comparing two groups at a single time point, an unpaired *t*-test was used. With the exception of offspring growth trajectory where the average body weight for each litter was calculated, only one male per litter was used in any one analysis. For all comparisons, *P* < .05 was considered statistically significant.

## Results

### High-sugar, high-fat feeding in females increases body weight and adiposity

Obesity was induced in female mice by feeding a high-fat, high-sugar diet 6 weeks prior to conception and during pregnancy and lactation. Control females were fed a standard laboratory chow throughout. Concurrent with previous results (12, 13), dams fed the highly palatable obesogenic diet were heavier before (33.3 ± 1.2 g [n = 12] vs 27.7 ± 0.7 g [n = 8], *P* < .01) and immediately after parturition (35.2 ± 0.9 g [n = 14] vs 32.4 ± 0.5 g [n = 16], *P* < .01), and at weaning (41.3 ± 1.6 g [n = 14] vs 33.5 ± 0.6 g [n = 16], *P* < .001) compared with control dams. Fat and lean mass was measured in dams at weaning by time-domain nuclear magnetic resonance. Both absolute (14.3 ± 1.6 g vs 3.4 ± 0.3 g, *P* < .001) and relative fat mass as a percentage of body weight (34.5 ± 2.7 % vs 12.1 ± 0.7 %, *P* < .001) were increased in dams fed the obesogenic diet. However, absolute lean mass was similar between groups (control dams, 19.8 ± 0.3 g vs obese dams, 19.8 ± 0.2 g).

### Exposure to maternal obesity increased heart weight and cardiomyoycte cell size independent of offspring body weight

**Figure 1.**
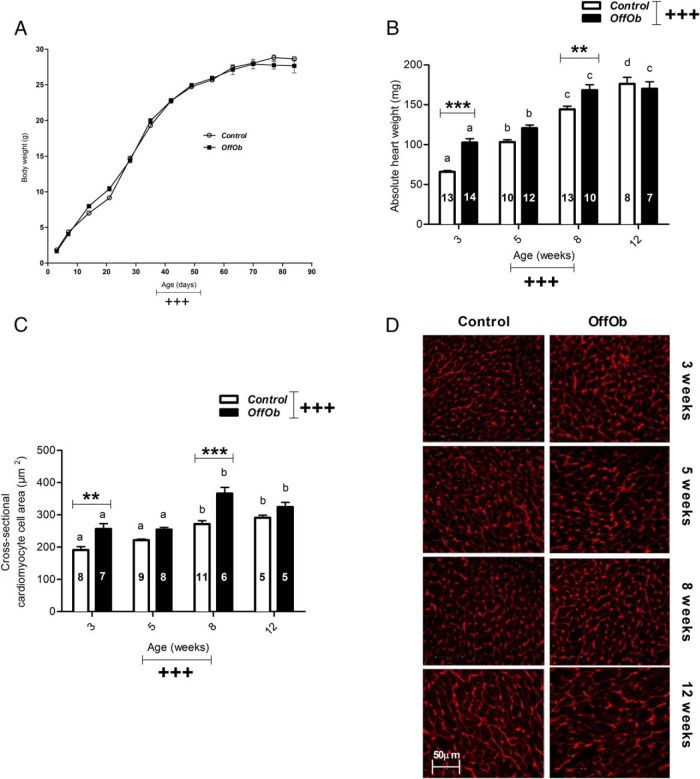
Offspring body weight and cardiac morphometry (3, 5, 8, and 12 wk of age). A, Growth trajectory. B, Absolute heart weight. C) Cross-sectional cardiomyocyte cell area. D) Representative images of cardiomyocyte cell borders stained with wheat germ agglutinin. Control offspring (○ or *white bars*) and OffOb offspring (■ or *black bars*). For offspring growth trajectory from day 3–21, controls, n = 31; OffOb, n = 38. From day 21–35, controls, n = 29; OffOb, n = 26. From day 35–56, controls, n = 20; OffOb, n = 20. From day 56–84, controls, n = 9; OffOb, n = 7. Sample number is represented inside the histogram and data are presented as mean ± SEM. Effect of maternal diet and age is marked as +++, *P* < .001. Between-group comparisons are represented by asterisks, **, *P* < .01; ***, *P* < .001. For within-group comparisons, different letters are significantly different from each other (*P* < .05).

There was no effect of maternal diet on offspring growth trajectory from 3–12 weeks of age (Figure 1A). Also, there were no differences in retroperitoneal (controls, 83 ± 19 mg vs OffOb, 83 ± 15 mg; n = 8 and n = 7 per group, respectively) and epididymal fat pad weights (controls, 373 ± 46 mg vs OffOb 448 ± 57 mg; n = 8 and n = 7 per group, respectively) between groups at 12 weeks of age when cardiac function was assessed. Furthermore, there was no overall effect of maternal diet on circulating factors including total cholesterol, triglycerides, and free fatty acids (Table 1).

**Table 1.** Circulating Factors in Offspring at 3, 5, and 8 Weeks of Age

	Week 3		Week 5		Week 8	
	Control	OffOb	Control	OffOb	Control	OffOb
Total cholesterol, mmol/L^a^	2.44 ± 0.15	2.62 ± 0.40	3.75 ± 0.09	3.42 ± 0.22	3.22 ± 0.12	3.33 ± 0.12
Triglycerides, mmol/L	0.78 ± 0.10	1.20 ± 0.07	0.88 ± 0.05	1.83 ± 0.74	1.73 ± 0.18	1.58 ± 0.20
Free fatty acids, μmol/L^a^	947 ± 73	813 ± 87	1577 ± 175	1555 ± 200	878 ± 1	831 ± 90

a Significant overall effect of age. 3, 5, and 8 weeks of age; controls, n = 10; OffOb, n = 10.

Cardiac growth was accelerated in offspring of obese dams relative to controls with an effect of maternal diet on both absolute (Figure 1B) and relative heart weight (Table 2). The most pronounced differences were observed at 3 and 8 weeks of age. However, by 12 weeks, heart weights were similar in both groups.

**Table 2.** Cardiac Parameters in Offspring at 3, 5, 8, and 12 Weeks of Age

Measurements	Week 3		Week 5		Week 8		Week 12	
		n		n		n		n
Heart weight:body weight^a,b^								
Control	0.65 ± 0.02	12	0.65 ± 0.02	10	0.61 ± 0.02	12	0.62 ± 0.03	8
OffOb	1.05 ± 0.04***	12	0.74 ± 0.03	12	0.73 ± 0.03*	10	0.62 ± 0.02	7
LV volume (mm^3^)^a,b^								
Control	14.33 ± 0.88	9	27.77 ± 0.61	8	28.53 ± 1.32	8	29.54 ± 2.49	9
OffOb	21.53 ± 1.03*	8	34.66 ± 1.64*	8	32.80 ± 2.03	7	32.67 ± 0.59	7
RV volume (mm^3^)^a,b^								
Control	2.07 ± 0.08	9	2.95 ± 0.10	8	7.06 ± 0.41	8	6.33 ± 0.63	9
OffOb	2.81 ± 0.18	8	3.52 ± 0.10*	8	6.50 ± 0.27	7	7.29 ± 0.54	7

a Significant overall effect of maternal diet.

b Significant overall effect of age.

Between-group comparisons are represented by asterisks,

* , *P* < .05 and

*** , *P* < .001.

To address whether the increased heart weight in offspring of obese dams was a consequence of cardiac remodelling, nonbiased cardiac stereology was performed to assess both ventricular wall and chamber volumes. Both LV and RV volumes were increased in offspring exposed to maternal obesity (Table 2). Because there was evidence of ventricular wall thickening, particularly in the LV, cell-specific hypertrophy was addressed. Frozen sections from the LV were stained with wheat germ agglutinin to highlight the cardiomyocyte cell borders. Cell area was quantified using computer-assisted software. In line with ventricular wall thickening, cardiomyocyte cell hypertrophy was observed in offspring of obese dams (Figure 1, C and D). The increase in cell size was most pronounced at 3 and 8 weeks of age, however these differences were no longer apparent by 12 weeks of age.

### Re-expression of fetal genes indicative of pathologic cardiac hypertrophy was observed in offspring of obese dams

**Figure 2.**
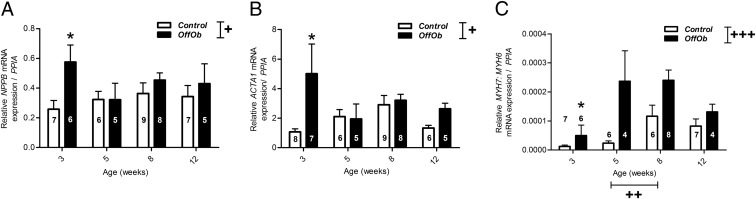
Offspring fetal gene expression (relative mRNA levels normalized to *PPIA*) at 3, 5, 8, and 12 wk of age. A, *NPPB*; B, *ACTA1*; C, *MYH7:MYH6*. Control offspring (*white bars*) and OffOb offspring (*black bars*). Sample number is represented inside the histogram and data are presented as mean ± SEM. Overall effect of maternal diet and age is signified by, +, *P* < .05; ++, *P* < .01; +++, *P* < .001. Between-group comparisons are represented by asterisks, *, *P* < .05.

We further investigated whether the observed cardiac hypertrophy was physiologic or pathologic. Cardiac fetal genes (*NPPA*, *NPPB*, *MYH6*, *MYH7* and *ACTA1*) are usually only expressed during fetal life and re-expression of these genes in adult life is an indicator of pathologic cardiac hypertrophy (26). Increased mRNA expression of cardiac *NPPB* (*P* < .05) and *ACTA1* (*P* < .05) was observed in offspring of obese dams relative to controls (Figure 2, A and B). In the healthy mouse heart, expression of *MYH6* predominates over *MYH7* (27, 28); however, during periods of cardiac stress there is a switch in ratio of these two genes, whereby the energy-conservative β-myosin heavy chain becomes more highly expressed. Our results demonstrated an increase in ratio of *MHY7:MYH6* in offspring of obese dams from weaning (Figure 2C). There is evidence that this switch can negatively affect cardiac function (29).

### Baseline cardiac function is impaired in offspring of obese dams by young adulthood with evidence of sympathetic dominance, a pre-eminent marker of heart failure

**Figure 3.**
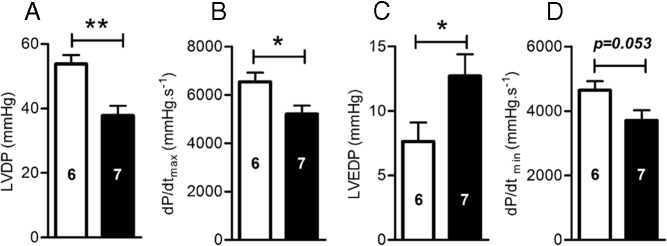
Offspring baseline cardiac function at 12 wk of age. A, LVDP; B, dP/d*t*_max_; C, LVEDP. D) dP/d*t*_min_. Control offspring (*white bars*) and OffOb offspring (*black bars*). Sample number is represented inside the histogram and data are presented as mean ± SEM, *, *P* < .05; **, *P* < .01.

Confronted with evidence of pathologic cardiac hypertrophy at the structural and molecular levels in offspring of obese dams, we assessed the potential effect on cardiac function. This was measured in 12-week old offspring ex vivo using the isolated Langendorff heart perfusion technique. Hearts from offspring of obese dams had systolic dysfunction including reduced LV developed pressure (LVDP) (*P* < .01) and an impaired velocity of contraction (dP/d*t*_max_) (*P* < .05) relative to controls (Figure 3, A and B). Hearts of offspring from obese pregnancy also showed increased LV end diastolic pressure (LVEDP) (*P* < .05), (Figure 3C), a well-established marker of diastolic dysfunction (30). Furthermore, ventricular relaxation was impaired in offspring of obese dams, with a trend observed toward a lower velocity of relaxation (dP/d*t*_min_, *P* = .053), (Figure 3D).

**Figure 4.**
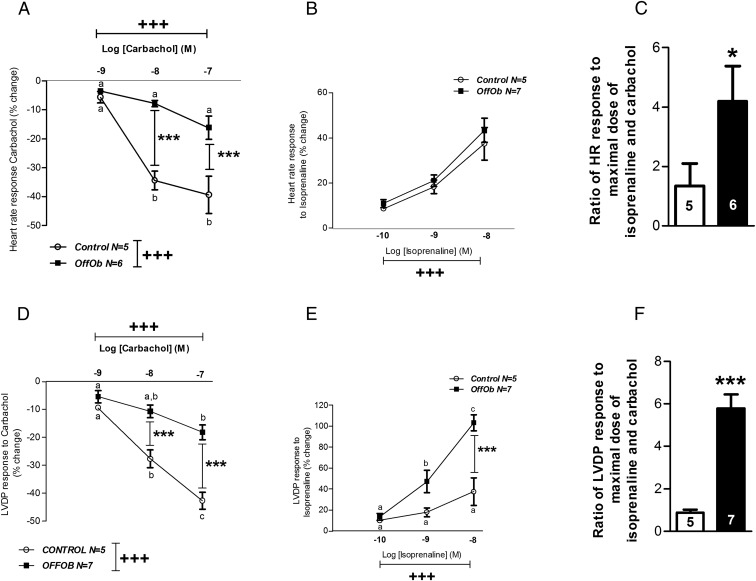
Chronotropic and inotropic response to increasing doses of carbachol (10^−9^–10^−7^ M) and isoprenaline (10^−10^–10^−8^ M) in offspring at 12 wk of age. A and B, Chronotropic response. C, Sympathetic:parasympathetic ratio for HR. D and E, Inotropic response. F, Sympathetic:parasympathetic ratio for LVDP. Control offspring (○ or *white bars*) and OffOb offspring (■ or *black bars*). Sample number is represented inside the histogram or on the figure legend, and data are presented as mean ± SEM. Overall effect of maternal diet and dose is signified by, +++, *P* < .001. Between-group comparisons are represented by asterisks, *, *P* < .05; ***, *P* < .001. For within-group comparisons, different letters are significantly different from each other (*P* < .05).

The isolated Langendorff heart perfusion technique also allowed us to measure cardiac responsiveness (chronotropic and inotropic) to parasympathetic and sympathetic agents such as carbachol and isoprenaline, respectively. The response to these drugs was assessed in a dose-responsive manner. Hearts from offspring of obese dams had a blunted chronotropic response to carbachol (*P* < .001), (Figure 4A). However, there was no difference between groups on the chronotropic response to isoprenaline (Figure 4B). When expressed as a ratio of sympathetic to parasympathetic chronotropic responses, hearts from offspring of obese dams showed sympathetic dominance (*P* < .05), (Figure 4C). These hearts also demonstrated an attenuated inotropic response to carbachol compared with controls (*P* < .001), (Figure 4D). However, administration of isoprenaline enhanced the inotropic response in offspring of obese dams (*P* < .001), (Figure 4E). Hence, the ratio of sympathetic to parasympathetic inotropic responses was also markedly elevated compared with controls (*P* < .001), (Figure 4F). These characteristics define cardiac sympathetic dominance in offspring of obese dams in relation to both the inotropic and chronotropic response. It is therefore significant that long-term cardiac activation by the sympathetic nervous system is considered a surrogate marker of heart failure (31) as this suggests that an overt sympathetic response provides evidence that the hearts of these offspring are failing.

### Cardiac dysfunction and sympathetic dominance in offspring of obese dams could be explained by the down-regulation of SERCA2a and up-regulation of β_1_ adrenergic receptors

**Figure 5.**
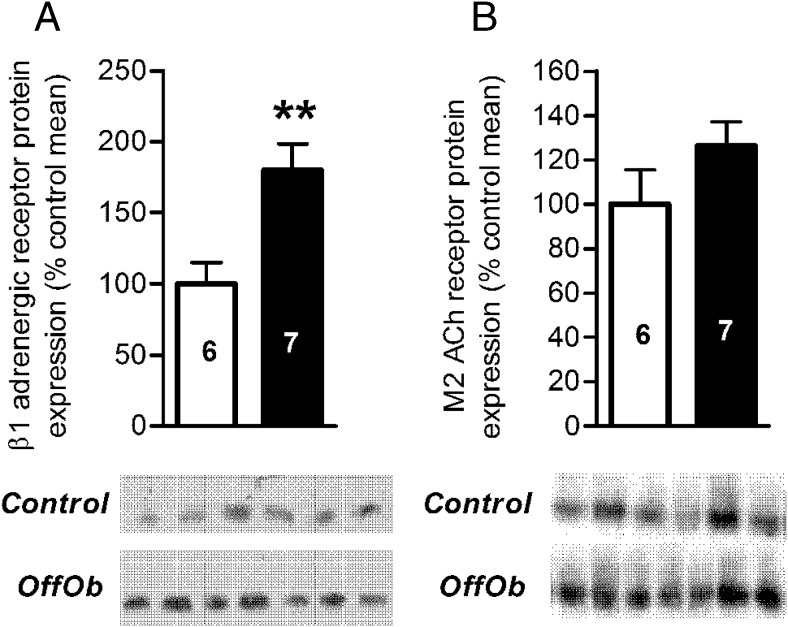
Protein expression of receptors expressed relative to control offspring. A) β_1_ adrenergic receptor (50 kDa). B) Muscarinic type-2 acetylcholine receptor (64 kDa). Control offspring (*white bars*) and OffOb offspring (*black bars*). Sample number is represented inside the histogram and data are presented as mean ± SEM, **, *P* < .01. Blots shown below the histograms are from the same gel.

To address the possible cause of both the sympathetic dominance and cardiac dysfunction, proteins involved in the cardiac contractile machinery, diastolic function, and sympathetic tone were measured in offspring at 12 weeks of age. β_1_ adrenergic receptors are the most predominantly expressed subtype in the murine heart (32). Furthermore, they have an important role in both the chronotropic and inotropic response to sympathetic stimulation (33, 34) and are involved in calcium mobilization. Concurrent with the functional sympathetic dominance, protein levels of the β_1_ adrenergic receptor were increased in hearts of offspring of obese dams (Figure 5A). However, the diminished parasympathetic response in offspring of obese dams was not accompanied by differences in muscarinic type 2-acetylcholine receptor levels (Figure 5B), the predominant subtype associated with the parasympathetic response in the heart.

**Figure 6.**
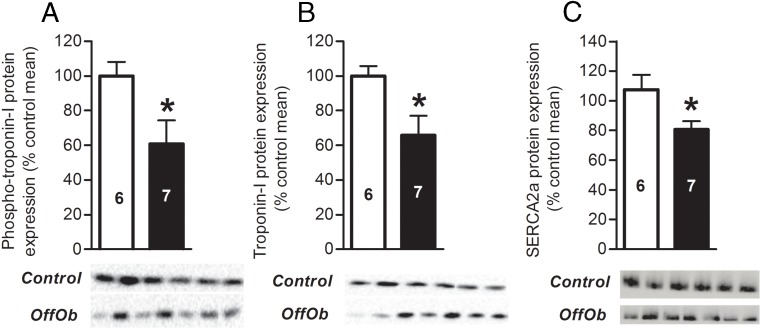
Protein expression of contractile proteins expressed relative to control offspring. A) Phospho-troponin-I (serine 23/24) (28 kDa). B) Total troponin-I (29 kDa). C) SERCA2a (114 kDa). Control offspring (*white bars*) and OffOb offspring (*black bars*). Sample number is represented inside the histogram and data are presented as mean ± SEM, *, *P* < .05. Blots shown below the histograms are from the same gel.

Defects in cardiac myofilaments are also known to have a role in cardiac disease (35, 36). Cardiac phosphorylated troponin-I (serine 23/24) (*P* < .05) and total troponin-I (*P* < .05) were significantly reduced in offspring from obese dams (Figure 6, A and B). In a cardiac-specific troponin-I knockout mouse model, loss of troponin-I was compensated for by its fetal isoform up to 15 days of age. However, long-term compensation was not maintained and mice developed heart failure at 18 days of age (37). This highlights the importance of troponin-I for cardiac function and suggests that a reduction of troponin-I in offspring of obese dams may impair cardiac function. However the ratio of phosphorylated to total troponin-I between groups was not different (controls, 100 ± 19% control mean vs OffOb 90 ± 23% control mean; *P* = .74). There was also no difference in the protein levels of tropomyosin (controls, 100 ± 11% control mean vs OffOb 64 ± 17% control mean; *P* = .13). Sarco/endoplasmic reticulum Ca^2+^ ATPase (SERCA2a) is important for extrusion of cytosolic calcium necessary for cardiomyocyte relaxation as well as in accumulating calcium in the sarcoplasmic reticulum in preparation for contraction (38). Protein levels of SERCA2a were attenuated (*P* < .05) in offspring of obese dams (Figure 6C). This provides a mechanism for both impaired systolic and diastolic function in these offspring.

## Discussion

Our study has shown that exposure to maternal diet-induced obesity during pregnancy and lactation leads to cardiac hypertrophy and dysfunction in young adult mouse offspring, independent of offspring body weight and adiposity at 12 weeks of age. It also clearly demonstrates that cardiac hypertrophy is a very early consequence of maternal diet-induced obesity, apparent from as early as weaning and accompanied by substantial cardiac dysfunction.

Pathologic cardiac hypertrophy is often characterized by re-expression of genes usually only expressed during fetal life (39). There was evidence of re-expression of such genes in offspring of obese dams compared with controls. *NPPB* encodes brain natriuretic peptide (BNP) and is a well-established serum marker used in the prognosis of heart failure in the clinic (40, 41). BNP is a vasoactive peptide released with the aim of reducing blood pressure and/or volume in response to an increase in cardiac muscle stretch (42). The increased levels of *NPPB* transcript in offspring of obese dams, particularly at 3 weeks of age suggests a compensatory response to increased cardiomyocyte muscle stretch. Re-expression of *ACTA1* that encodes α-smooth muscle actin has been associated with cardiac hypertrophy in vitro and in vivo, in animal models and humans (43, 44). Increased transcript levels of *ACTA1* was seen at 3 and 12 weeks of age, with the greatest difference observed at 3 weeks of age. Elevated α-smooth muscle actin is associated with a rapid increase in heart volume and positively correlates with cardiac contractility in humans and animal models (44, 45). Therefore, increased *ACTA1* acts as a compensatory mechanism to increase the contractile capacity of the heart in response to an increase in volume (46). This could be the mechanism adopted in the hearts of offspring of obese dams, whereby the greatest increase in *ACTA1* transcript was observed at 3 weeks, the age at which there was the greatest difference in heart weight between groups. In the mouse heart there is a predominance of α-myosin heavy chain (MHC), encoded by the *MYH6* gene, and is known as the fast twitch fiber due to its higher ATPase capacity (27, 28, 47). By 3 weeks of age there was a switch in the ratio of MHC to favor the slow twitch β-MHC in offspring of obese dams. A switch in the ratio of these isoforms could act to preserve energy in response to pathologic heart stress; however, although initially conserving energy, a long-term switch is associated with cardiac dysfunction (28, 48). Changes in the ratio of MHC are directly proportional to the performance and efficiency of the heart (49); therefore, these changes are consistent with the observed cardiac dysfunction in the offspring of obese dams.

Offspring of obese dams showed systolic and diastolic dysfunction, with evidence of impaired ventricular contractility and reduced myocardial compliance. SERCA2a removes cytosolic calcium required for cardiomyoycte relaxation, while accumulating calcium in the sarcoplasmic reticulum in preparation for contraction. Therefore, reduced SERCA2a could explain both the systolic and diastolic dysfunction in offspring of obese dams. Reduced expression of SERCA2a has been associated with cardiac dysfunction, ischemia-reperfusion injury, and heart failure (50–52). Furthermore, these offspring also showed reduced protein expression of total troponin-I that plays an important role in normal cardiac function. In a cardiac-specific knockout mouse model, loss of adult troponin-I was initially compensated for by the fetal isoform. However, long-term compensation was not maintained and mice developed heart failure (37). Differences in the phosphorylation of myofilament troponin-I have been observed between healthy and failing hearts (53, 54). Whereas absolute phosphorylation levels of troponin-I (serine 23/24) were reduced in offspring of obese dams, there was no difference between groups when normalized to total levels.

In offspring of obese dams, systolic and diastolic dysfunction was associated with cardiac sympathetic dominance. Elevated LV end diastolic pressure is itself associated with increased mortality (55). Cardiac sympathetic dominance is adaptive and aimed at maintaining cardiac output. However, heightened cardiac sympathetic excitation coupled with diminished cardiac parasympathetic reactivity have been associated with CVD, being an unsustainable condition, and leading to eventual heart failure (31, 56). The increased sympathetic dominance in the offspring of obese dams can be explained by up-regulation of β_1_ adrenergic receptors. The etiology underlying the attenuated parasympathetic responsiveness in the hearts of offspring of obese dams may be receptor independent given that there were no differences in the protein expression of the predominant muscarinic (M2) acetylcholine receptor between groups. This may suggest a potential role of effectors downstream of the M2 acetylcholine receptor including G_i_ activity, adenylyl cylase, phospholipase C, potassium channels, and protein tyrosine kinases [reviewed in (57)].

Notably, cardiac dysfunction was present at 12 weeks of age when the cellular and structural phenotype of cardiac hypertrophy was no longer apparent. Although cardiac hypertrophy acts as a protective mechanism, it is well understood that this phenotype cannot be maintained long-term. Therefore, we hypothesize that at 12 weeks of age cardiac hypertrophy is subsiding because the heart is no longer able to sustain adequate cardiac function. Based on the Langendorff data that showed cardiac sympathetic dominance in offspring of obese dams (a pre-eminent marker of heart failure) with age (31), these offspring may be on the pathway to premature heart failure. The potential for heart failure in these offspring is supported by the Langendorff data, which show impaired systolic function and LV stiffening in young adulthood.

Potential underlying mechanisms through which cardiac hypertrophy and cardiac dysfunction may arise have been suggested by previous molecular studies. One study, using the same mouse model, showed that hyperinsulinemia in offspring of obese dams was associated with increased action through both the phosphoinositide 3 kinase and mitogen-activated protein kinase pathways in cardiac tissue (13). Both of these pathways have been associated with cardiac hypertrophy. Furthermore, offspring of obese dams had increased p38MAPK, a marker of pathological cardiac hypertrophy (58). Oxidative stress has also been implicated in a variety of programming models as a potential mechanism underlying developmental programming in the offspring. Offspring of obese dams at 8 weeks of age had increased lipid peroxidation, impaired antioxidant capacity, and an increased potential for the generation of superoxide (13). A model of maternal hypoxia in rodents has also been associated with enhanced oxidative stress in both the fetal heart and vasculature at 4 months of age (23). Furthermore, the adverse effects of maternal hypoxia on the offspring cardiovascular system were prevented by maternal treatment with the antioxidant Vitamin C (23). In a sheep model of maternal overnutrition, increased levels of oxidative stress were found in fetal tissues accompanied by an impaired response to workload stress (15). Oxidative stress has therefore been implicated in a variety of developmental programming models, suggesting that it may be a common mechanism underlying the programming of CVD by a range of suboptimal early life exposures.

We conclude that cardiac hypertrophy is a very early consequence of maternal diet-induced obesity that is associated with impaired systolic and diastolic function in young-adult offspring of obese dams. Importantly, these changes were independent of differences in offspring body weight. These findings therefore provide a potential mechanistic basis to explain recent human observations linking maternal obesity to premature death from CVD. In light of the growing epidemic of obesity worldwide, these findings have major implications for the long-term cardiovascular health of children today. Furthermore, understanding of the mechanisms mediating the effects of maternal obesity on offspring cardiac function is therefore urgently needed.

## References

[1] Heslehurst N Rankin J Wilkinson JR Summerbell CD A nationally representative study of maternal obesity in England, UK: Trends in incidence and demographic inequalities in 619 323 births, 1989–2007. Int J Obes (Lond). 2010;34(3):420–428. 20029373 10.1038/ijo.2009.250

[2] Crane JM Murphy P Burrage L Hutchens D Maternal and perinatal outcomes of extreme obesity in pregnancy. J Obstet Gynaecol Can. 2013;35(7):606–611. 23876637 10.1016/S1701-2163(15)30879-3

[3] Athukorala C Rumbold AR Willson KJ Crowther CA The risk of adverse pregnancy outcomes in women who are overweight or obese. BMC Pregnancy Childbirth. 2010;10:56. 20849609 10.1186/1471-2393-10-56 PMC2949787

[4] Gluckman PD Hanson MA Cooper C Thornburg KL Effect of in utero and early-life conditions on adult health and disease. N Engl J Med. 2008;359(1):61–73. 18596274 10.1056/NEJMra0708473 PMC3923653

[5] Giussani DA Davidge ST Developmental programming of cardiovascular disease by prenatal hypoxia. J Dev Orig Health Dis. 2013;6(5):328–337. 24970726 10.1017/S204017441300010X

[6] Reynolds RM Allan KM Raja EA Maternal obesity during pregnancy and premature mortality from cardiovascular event in adult offspring: Follow-up of 1 323 275 person years. BMJ. 2013;347:f4539. 23943697 10.1136/bmj.f4539 PMC3805484

[7] Forsén T Eriksson JG Tuomilehto J Teramo K Osmond C Barker DJ Mother's weight in pregnancy and coronary heart disease in a cohort of Finnish men: Follow up study. BMJ. 1997;315(7112):837–840. 9353502 10.1136/bmj.315.7112.837 PMC2127571

[8] Liang C Oest ME Prater MR Intrauterine exposure to high saturated fat diet elevates risk of adult-onset chronic diseases in C57BL/6 mice. Birth Defects Res B Dev Reprod Toxicol. 2009;86(5):377–384. 19750488 10.1002/bdrb.20206

[9] Guberman C Jellyman JK Han G Ross MG Desai M Maternal high-fat diet programs rat offspring hypertension and activates the adipose renin-angiotensin system. Am J Obstet Gynecol. 2013;209(3):262.e261–e268. 23743273 10.1016/j.ajog.2013.05.023 PMC4010310

[10] Ghosh P Bitsanis D Ghebremeskel K Crawford MA Poston L Abnormal aortic fatty acid composition and small artery function in offspring of rats fed a high fat diet in pregnancy. J Physiol. 2001;533(Pt 3):815–822. 11410637 10.1111/j.1469-7793.2001.00815.x PMC2278671

[11] Bringhenti I Moraes-Teixeira JA Cunha MR Maternal obesity during the preconception and early life periods alters pancreatic development in early and adult life in male mouse offspring. PLoS One. 2013;8(1):e55711. 23383269 10.1371/journal.pone.0055711 PMC3561327

[12] Samuelsson AM Matthews PA Argenton M Diet-induced obesity in female mice leads to offspring hyperphagia, adiposity, hypertension, and insulin resistance: A novel murine model of developmental programming. Hypertension. 2008;51(2):383–392. 18086952 10.1161/HYPERTENSIONAHA.107.101477

[13] Fernandez-Twinn DS Blackmore HL Siggens L The programming of cardiac hypertrophy in the offspring by maternal obesity is associated with hyperinsulinemia, AKT, ERK, and mTOR activation. Endocrinology. 2012;153(12):5961–5971. 23070543 10.1210/en.2012-1508 PMC3568261

[14] Fan X Turdi S Ford SP Influence of gestational overfeeding on cardiac morphometry and hypertrophic protein markers in fetal sheep. J Nutr Biochem. 2011;22(1):30–37. 20188535 10.1016/j.jnutbio.2009.11.006 PMC2901772

[15] Wang J Ma H Tong C Overnutrition and maternal obesity in sheep pregnancy alter the JNK-IRS-1 signaling cascades and cardiac function in the fetal heart. FASEB J. 2010;24(6):2066–2076. 20110268 10.1096/fj.09-142315 PMC2874473

[16] Han J Xu J Epstein PN Liu YQ Long-term effect of maternal obesity on pancreatic beta cells of offspring: Reduced beta cell adaptation to high glucose and high-fat diet challenges in adult female mouse offspring. Diabetologia. 2005;48(9):1810–1818. 16010523 10.1007/s00125-005-1854-8

[17] Fan L Lindsley SR Comstock SM Maternal high-fat diet impacts endothelial function in nonhuman primate offspring. Int J Obes (Lond). 2013;37(2):254–262. 22450853 10.1038/ijo.2012.42 PMC3468685

[18] Moreira AS Teixeira Teixeira M da Silveira Osso F Left ventricular hypertrophy induced by overnutrition early in life. Nutr Metab Cardiovasc Dis. 2009;19(11):805–810. 19359151 10.1016/j.numecd.2009.01.008

[19] Elahi MM Cagampang FR Mukhtar D Anthony FW Ohri SK Hanson MA Long-term maternal high-fat feeding from weaning through pregnancy and lactation predisposes offspring to hypertension, raised plasma lipids and fatty liver in mice. Br J Nutr. 2009;102(4):514–519. 19203419 10.1017/S000711450820749X

[20] Samuelsson AM Morris A Igosheva N Evidence for sympathetic origins of hypertension in juvenile offspring of obese rats. Hypertension. 2010;55(1):76–82. 19901159 10.1161/HYPERTENSIONAHA.109.139402

[21] Artham SM Lavie CJ Milani RV Ventura HO Obesity and hypertension, heart failure, and coronary heart disease-risk factor, paradox, and recommendations for weight loss. Ochsner J. 2009;9(3):124–132. 21603427 PMC3096264

[22] Hubert HB Feinleib M McNamara PM Castelli WP Obesity as an independent risk factor for cardiovascular disease: A 26-year follow-up of participants in the Framingham Heart Study. Circulation. 1983;67(5):968–977. 6219830 10.1161/01.cir.67.5.968

[23] Giussani DA Camm EJ Niu Y Developmental programming of cardiovascular dysfunction by prenatal hypoxia and oxidative stress. PLoS One. 2012;7(2):e31017. 22348036 10.1371/journal.pone.0031017 PMC3278440

[24] Chelu MG Sarma S Sood S Calmodulin kinase II-mediated sarcoplasmic reticulum Ca2+ leak promotes atrial fibrillation in mice. J Clin Invest. 2009;119(7):1940–1951. 19603549 10.1172/JCI37059 PMC2701862

[25] Niu Y Herrera EA Evans RD Giussani DA Antioxidant treatment improves neonatal survival and prevents impaired cardiac function at adulthood following neonatal glucocorticoid therapy. J Physiol. 2013;591(Pt 20):5083–5093. 23940378 10.1113/jphysiol.2013.258210 PMC3810811

[26] Kuwahara K Nishikimi T Nakao K Transcriptional regulation of the fetal cardiac gene program. J Pharmacol Sci. 2012;119(3):198–203. 22786561 10.1254/jphs.12r04cp

[27] Ng WA Grupp IL Subramaniam A Robbins J Cardiac myosin heavy chain mRNA expression and myocardial function in the mouse heart. Circ Res. 1991;68(6):1742–1750. 2036722 10.1161/01.res.68.6.1742

[28] Krenz M Robbins J Impact of beta-myosin heavy chain expression on cardiac function during stress. J Am Coll Cardiol. 2004;44(12):2390–2397. 15607403 10.1016/j.jacc.2004.09.044

[29] Tardiff JC Hewett TE Factor SM Vikstrom KL Robbins J Leinwand LA Expression of the beta (slow)-isoform of MHC in the adult mouse heart causes dominant-negative functional effects. Am J Physiol Heart Circ Physiol. 2000;278(2):H412–419. 10666070 10.1152/ajpheart.2000.278.2.H412

[30] Sakata Y Ohtani T Takeda Y Yamamoto K Mano T Left ventricular stiffening as therapeutic target for heart failure with preserved ejection fraction. Circ J. 2013;77(4):886–892. 23486165 10.1253/circj.cj-13-0214

[31] Kaye DM Lefkovits J Jennings GL Bergin P Broughton A Esler MD Adverse consequences of high sympathetic nervous activity in the failing human heart. J Am Coll Cardiol. 1995;26(5):1257–1263. 7594040 10.1016/0735-1097(95)00332-0

[32] Woo AY Xiao RP β-Adrenergic receptor subtype signaling in heart: From bench to bedside. Acta Pharmacol Sin. 2012;33(3):335–341. 22286918 10.1038/aps.2011.201 PMC4077138

[33] Philipp M Hein L Adrenergic receptor knockout mice: Distinct functions of 9 receptor subtypes. Pharmacol Ther. 2004;101(1):65–74. 14729393 10.1016/j.pharmthera.2003.10.004

[34] Rohrer DK Desai KH Jasper JR Targeted disruption of the mouse beta1-adrenergic receptor gene: Developmental and cardiovascular effects. Proc Natl Acad Sci U S A. 1996;93(14):7375–7380. 8693001 10.1073/pnas.93.14.7375 PMC38992

[35] de Tombe PP Cardiac myofilaments: Mechanics and regulation. J Biomech. 2003;36(5):721–730. 12695002 10.1016/s0021-9290(02)00450-5

[36] Day SM Westfall MV Metzger JM Tuning cardiac performance in ischemic heart disease and failure by modulating myofilament function. J Mol Med (Berl). 2007;85(9):911–921. 17396243 10.1007/s00109-007-0181-6

[37] Huang X Pi Y Lee KJ Cardiac troponin I gene knockout: A mouse model of myocardial troponin I deficiency. Circ Res. 1999;84(1):1–8. 9915769 10.1161/01.res.84.1.1

[38] Kawase Y Hajjar RJ The cardiac sarcoplasmic/endoplasmic reticulum calcium ATPase: A potent target for cardiovascular diseases. Nat Clin Pract Cardiovasc Med. 2008;5(9):554–565. 18665137 10.1038/ncpcardio1301

[39] Distefano G Sciacca P Molecular pathogenesis of myocardial remodeling and new potential therapeutic targets in chronic heart failure. Ital J Pediatr. 2012;38:41. 22971785 10.1186/1824-7288-38-41 PMC3480957

[40] McCullough PA Nowak RM McCord J B-type natriuretic peptide and clinical judgment in emergency diagnosis of heart failure: Analysis from Breathing Not Properly (BNP) Multinational Study. Circulation. 2002;106(4):416–422. 12135939 10.1161/01.cir.0000025242.79963.4c

[41] Nishii M Inomata T Takehana H Prognostic utility of B-type natriuretic peptide assessment in stable low-risk outpatients with nonischemic cardiomyopathy after decompensated heart failure. J Am Coll Cardiol. 2008;51(24):2329–2335. 18549918 10.1016/j.jacc.2007.11.085

[42] Nishikimi T Maeda N Matsuoka H The role of natriuretic peptides in cardioprotection. Cardiovasc Res. 2006;69(2):318–328. 16289003 10.1016/j.cardiores.2005.10.001

[43] Bishopric NH Simpson PC Ordahl CP Induction of the skeletal alpha-actin gene in alpha 1-adrenoceptor-mediated hypertrophy of rat cardiac myocytes. J Clin Invest. 1987;80(4):1194–1199. 2821075 10.1172/JCI113179 PMC442365

[44] Adachi S Ito H Tamamori M Tanaka M Marumo F Hiroe M Skeletal and smooth muscle alpha-actin mRNA in endomyocardial biopsy samples of dilated cardiomyopathy patients. Life Sci. 1998;63(20):1779–1791. 9820122 10.1016/s0024-3205(98)00452-4

[45] Hewett TE Grupp IL Grupp G Robbins J Alpha-skeletal actin is associated with increased contractility in the mouse heart. Circ Res. 1994;74(4):740–746. 8137509 10.1161/01.res.74.4.740

[46] Clément S Chaponnier C Gabbiani G A subpopulation of cardiomyocytes expressing alpha-skeletal actin is identified by a specific polyclonal antibody. Circ Res. 1999;85(10):e51–58. 10559147 10.1161/01.res.85.10.e51

[47] Holubarsch C Goulette RP Litten RZ Martin BJ Mulieri LA Alpert NR The economy of isometric force development, myosin isoenzyme pattern and myofibrillar ATPase activity in normal and hypothyroid rat myocardium. Circ Res. 1985;56(1):78–86. 3155672 10.1161/01.res.56.1.78

[48] Chen Y Somji A Yu X Stelzer JE Altered in vivo left ventricular torsion and principal strains in hypothyroid rats. Am J Physiol Heart Circ Physiol. 2010;299(5):H1577–1587. 20729398 10.1152/ajpheart.00406.2010 PMC2993195

[49] Morkin E Regulation of myosin heavy chain genes in the heart. Circulation. 1993;87(5):1451–1460. 8490999 10.1161/01.cir.87.5.1451

[50] Somura F Izawa H Iwase M Reduced myocardial sarcoplasmic reticulum Ca(2+)-ATPase mRNA expression and biphasic force-frequency relations in patients with hypertrophic cardiomyopathy. Circulation. 2001;104(6):658–663. 11489771 10.1161/hc3101.093869

[51] Stüdeli R Jung S Mohacsi P Diastolic dysfunction in human cardiac allografts is related with reduced SERCA2a gene expression. Am J Transplant. 2006;6(4):775–782. 16539635 10.1111/j.1600-6143.2006.01241.x

[52] Talukder MA Kalyanasundaram A Zuo L Is reduced SERCA2a expression detrimental or beneficial to postischemic cardiac function and injury? Evidence from heterozygous SERCA2a knockout mice. Am J Physiol Heart Circ Physiol. 2008;294(3):H1426–1434. 18203847 10.1152/ajpheart.01016.2007

[53] Zakhary DR Moravec CS Stewart RW Bond M Protein kinase A (PKA)-dependent troponin-I phosphorylation and PKA regulatory subunits are decreased in human dilated cardiomyopathy. Circulation. 1999;99(4):505–510. 9927396 10.1161/01.cir.99.4.505

[54] van der Velden J Papp Z Zaremba R Increased Ca2+-sensitivity of the contractile apparatus in end-stage human heart failure results from altered phosphorylation of contractile proteins. Cardiovasc Res. 2003;57(1):37–47. 12504812 10.1016/s0008-6363(02)00606-5

[55] Salem R Denault AY Couture P Left ventricular end-diastolic pressure is a predictor of mortality in cardiac surgery independently of left ventricular ejection fraction. Br J Anaesth. 2006;97(3):292–297. 16835254 10.1093/bja/ael140

[56] Olshansky B Sabbah HN Hauptman PJ Colucci WS Parasympathetic nervous system and heart failure: Pathophysiology and potential implications for therapy. Circulation. 2008;118(8):863–871. 18711023 10.1161/CIRCULATIONAHA.107.760405

[57] Brodde OE Michel MC Adrenergic and muscarinic receptors in the human heart. Pharmacol Rev. 1999;51(4):651–690. 10581327

[58] Streicher JM Ren S Herschman H Wang Y MAPK-activated protein kinase-2 in cardiac hypertrophy and cyclooxygenase-2 regulation in heart. Circ Res. 2010;106(8):1434–1443. 20339119 10.1161/CIRCRESAHA.109.213199 PMC2903446

